# Supplementary material for: Host-Plant Selection Behavior of Ophraella communa, a Biocontrol Agent of the Invasive Common Ragweed Ambrosia artemisiifolia
Source: Insects. 2023 Mar 29;14(4):334. doi: 10.3390/insects14040334 (PMC10146365; doi:10.3390/insects14040334)
Supplement: Supplementary file 1 [file insects-14-00334-s001.zip › insects-2136368-supplementary.pdf]

Supplementary table S1. Three-way ANOVA of the effects of four plant species, days after release, and cages (blocks) on *Ophraella communa* distribution (adults and eggs) in outdoor cages. Pla refers to plant species, Day refers to days after release and blo refers to cages (blocks).

Factors	Adult		Egg	
	F	p	F	p
Pla	134.1	< 0.01	99.23	< 0.01
Day	2.186	0.124	29.91	0.033
Blo	2.719	0.274	2.992	0.471
Pla * Day	1.202	0.655	56.64	0.002
Pla * Blo	3.839	0.178	1.593	0.210
Day * Blo	2.966	0.357	1.429	0.863
Pla * Day * Blo	1.406	0.636	1.406	0.737

Supplementary table S2. Three-way ANOVA of the effects of plant species, distance from center, and ragweed cluster density on the number of *O. communa* individuals in different developmental stages on *A. artemisiifolia* and *H. annuus* planted. Pla refers to plant species, Dis refers to distance from center and Den refers to ragweed cluster density.

Years	Factors	Adult		Egg		Larva		Pupa	
		F	p	F	p	F	p	F	p
2010	Pla	544.8	< 0.01	520.7	< 0.01	1023.1	< 0.01	585.1	< 0.01
	Dis	0.304	0.738	2.201	0.117	1.546	0.219	0.202	0.817
	Den	0.932	0.398	0.675	0.512	0.256	0.775	1.297	0.279
	Pla * Dis	0.269	0.765	2.202	0.116	1.544	0.219	0.204	0.816
	Pla * Den	0.965	0.385	0.683	0.508	0.255	0.776	1.305	0.276
	Dis * Den	0.534	0.711	0.096	0.984	1.418	0.234	0.363	0.834
	Pla * Dis * Den	0.559	0.693	0.095	0.984	1.413	0.236	0.363	0.834
2011	Pla	520.1	< 0.01	1225	< 0.01	586.7	< 0.01	441.4	< 0.01
	Dis	1.626	0.202	1.461	0.237	0.665	0.517	0.305	0.738
	Den	0.029	0.972	0.148	0.863	0.946	0.392	1.711	0.186
	Pla * Dis	1.616	0.204	1.451	0.240	0.671	0.514	0.305	0.738
	Pla * Den	0.022	0.978	0.151	0.860	0.961	0.386	1.723	0.184
	Dis * Den	0.708	0.588	1.369	0.251	1.582	0.186	0.324	0.861
	Pla * Dis * Den	0.685	0.604	1.366	0.252	1.563	0.191	0.324	0.861

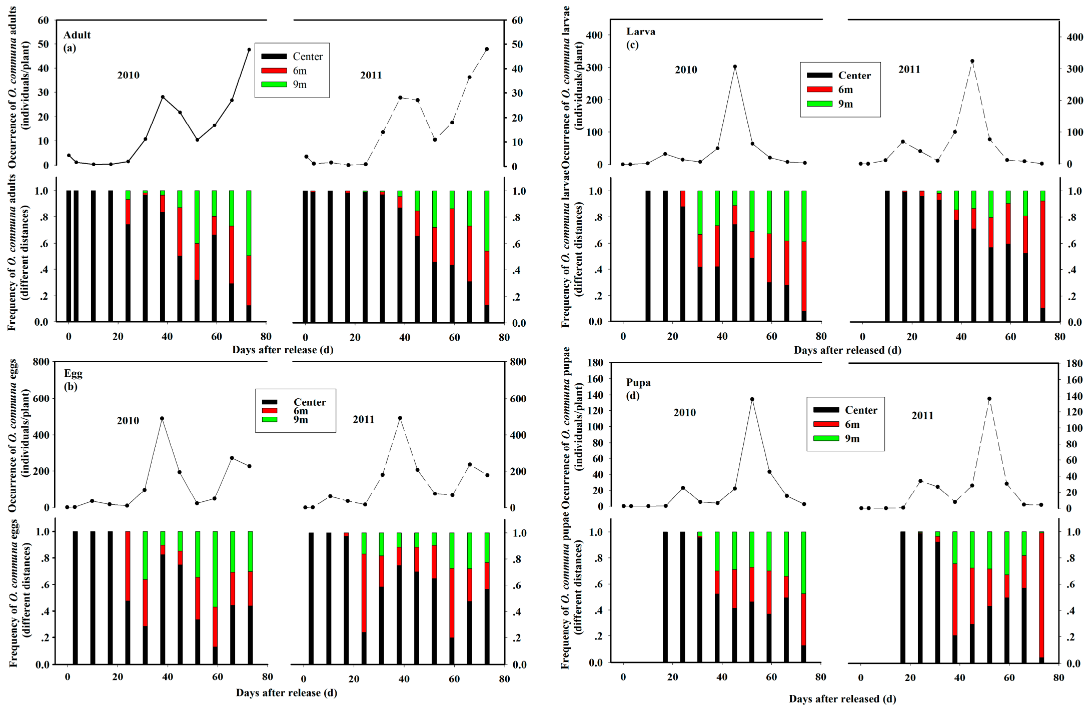

Figure S1. Occurrence and frequency of *Ophraella communa* individuals in different developmental stages on *A. artemisiifolia* planted at different distances. Centre indicates that *A. artemisiifolia* were planted in the center. *A. artemisiifolia* were planted in homocentric rings with a radius of 6 m and 9 m.
